# Prevalence and socio-demographic correlates of stunting and thinness among Pakistani primary school children

**DOI:** 10.1186/1471-2458-11-790

**Published:** 2011-10-11

**Authors:** Muhammad Umair Mushtaq, Sibgha Gull, Usman Khurshid, Ubeera Shahid, Mushtaq Ahmad Shad, Arif Mahmood Siddiqui

**Affiliations:** 1 Ubeera Memorial Research Society, Allama Iqbal Medical College, Lahore, 54000 Punjab, Pakistan; 2 District Health Office Nankana Sahib, Punjab Department of Health, Nankana Sahib, 39100 Punjab, Pakistan

## Abstract

**Background:**

Child growth is internationally recognized as an important indicator of nutritional status and health in populations. Child under-nutrition is estimated to be the largest contributor to global burden of disease, and it clusters in South Asia but literature on under-nutrition among school-aged children is difficult to find in this region. The study aimed to assess the prevalence and socio-demographic correlates of stunting and thinness among Pakistani primary school children.

**Methods:**

A population-based cross-sectional study was conducted with a representative multistage cluster sample of 1860 children aged 5-12 years in Lahore, Pakistan. Stunting (< -2 SD of height-for-age z-score) and thinness (< -2 SD of BMI-for-age z-score) were defined using the World Health Organization reference 2007. Chi-square test was used as the test of trend. Logistic regression was used to quantify the independent predictors of stunting and thinness and adjusted odds ratios (aOR) with 95% confidence interval (CI) were obtained. Linear regression was used to explore the independent determinants of height- and BMI-for-age z-scores. Statistical significance was considered at P < 0.05.

**Results:**

Eight percent (95% CI 6.9-9.4) children were stunted and 10% (95% CI 8.7-11.5) children were thin. Stunting and thinness were not significantly associated with gender. Prevalence of stunting significantly increased with age among both boys and girls (both P < 0.001) while thinness showed significant increasing trend with age among boys only (P = 0.034). Significant correlates of stunting included age > 8 years, rural area and urban area with low SES, low-income neighborhoods, lower parental education, more siblings, crowded housing and smoking in living place (all P < 0.001). Significant correlates of thinness included rural area and urban area with low SES, low-income neighborhoods and lower parental education (all P < 0.001), and age > 10 years (P = 0.003), more siblings (P = 0.016) and crowded housing (P = 0.006). In multivariate logistic regression analyses adjusted simultaneously for all factors, older age (aOR 3.60, 95% CI 1.89-6.88), urban area with low SES (aOR 2.58, 95% CI 1.15-5.81) and low-income neighborhoods (aOR 4.62, 95% CI 1.63-13.10) were associated with stunting while urban area with low SES (aOR 2.28, 95% CI 1.21-4.30) was associated with thinness. In linear regression analyses adjusted for all factors, low-income neighborhoods and older age were associated with lower height-for-age z-score while rural area with low/disadvantaged SES was associated with lower BMI-for-age z-score.

**Conclusions:**

Relatively low prevalence of stunting and thinness depicted an improvement in the nutritional status of school-aged children in Pakistan. However, the inequities between the poorest and the richest population groups were marked with significantly higher prevalence of stunting and thinness among the rural and the urban poor, the least educated, the residents of low-income neighborhoods and those having crowded houses. An increasing trend with age was observed in prevalence of stunting and thinness. Smoking in living place was associated with stunting. Findings suggest the need to implement evidence-based child health policy and strategies, prioritizing the poor and socially disadvantaged population.

## Background

Child growth is internationally recognized as an important indicator of nutritional status and health in populations. Stunting (low height-for-age) is acknowledged as the best indicator for child growth [1]. It indicates chronic under-nutrition and reflects the cumulative effects of under-nutrition and recurrent infections. Thinness (low BMI-for-age) corresponds to wasting and indicates acute under-nutrition, usually because of insufficient food intake or a high incidence of infectious diseases. Weight-for-age is inadequate indicator for monitoring child growth beyond pre-school years due to its inability to distinguish between relative height and body mass, therefore, BMI-for-age is recommended by the World Health Organization (WHO) and United States' Centers for Disease Control and Prevention (US CDC) to assess underweight in school-aged children and adolescents [2,3]. Stunting results from long-term nutritional deprivation, inadequate childcare and poor environmental and socio-cultural conditions. It is associated with higher morbidity and mortality, delayed mental development, poor educational achievement and reduced intellectual capacity, and is a strong predictor of human capital and social progress [4-6]. Child under-nutrition is estimated to be the largest contributor to global burden of disease, killing millions of children in the developing countries and causing heavy health expenditures [7-9]. It clusters in South Asia but literature on under-nutrition among school-aged children is difficult to find [10-13]. Routine surveillance for under-nutrition among school-aged children has not been conducted and national nutritional surveys do not include school-aged children in this region including Pakistan. In 2009-10, a cross-sectional survey titled the Nutritional Assessment among School-going Children in Lahore, Pakistan (NASCL) was conducted among primary school children aged 5-12 years. Prevalence and socio-demographic correlates of under-nutrition (stunting and thinness) among Pakistani primary school children is the subject of current paper.

## Methods

### Design, setting and sample

This was a population-based cross-sectional study among primary school children aged 5-12 years in Lahore, Pakistan. Lahore is the capital of Pakistan's most populous province Punjab and a metropolis with multiethnic populations. It has a population of about 9 million, including about 2.5 million primary school children aged 5-12 years, and 81% of the population resides in the urban area [14].

A multistage cluster sample of 1860 children aged 5-12 years in twelve primary schools of City District Lahore was enrolled. In the first stage, stratified random sampling based on the population and educational system characteristics, was used to have proportionate representation of gender, area of residence and socioeconomic status (SES). The list of all the public and private primary schools in Lahore was provided by the Punjab Department of Education. The listed schools were stratified according to the geographic area and monthly fee structure of the schools into following four strata: a) urban with high SES (urban area and fee > 2500 PKR), b) urban with middle SES (urban area and fee = 1000-2500 PKR), c) urban with low SES (urban area and fee < 1000 PKR), and d) rural with low/disadvantaged SES (rural area and fee ~100 PKR or free). The former two strata included private (including public-private mix) schools and the later two strata included public schools. In Pakistan, public schools cater low SES urban and rural children while high and middle SES urban children are educated in private and public-private mix schools. Three schools were selected at random from each stratum, and were contacted by the Departments of Education and Health to participate voluntarily in the study. School administration of three schools refused to participate and the next school was selected randomly from the respective stratum. For each school, a list of all classes in five grades (one to five) was obtained and one class in each grade was randomly selected. In this way, 60 classes, five from each school, were selected. For each of the selected classes, first 31 children on class attendance register, present on data collection day and aged 5-12 years, were included in the study. Children not willing to participate in the study were excluded. Sample size was calculated using Epi Info 6.04d (US CDC, 2004) with a confidence (1-α) of 95%, anticipated prevalence of 5% and margin of error of ± 1. The minimum sample size calculated was 1823 and a sample of 1860 was deemed sufficient.

### Data Collection

The sampled schools were visited on pre-arranged dates in summer 2009. Twenty trained senior medical students including 10 males and 10 females, lead by the Principal Investigator, collected the data. Data collection activity in each school was completed in two working days and it took four weeks to complete data collection. Data collection activity was planned to avoid measurements during the first two weeks of a new school term or immediately after a major holiday. Health education of children and teachers was also carried out after data collection in the respective school. Analogue physician health scales were used to measure height and weight [15]. All instruments were standardized before the measurements and balances were zero calibrated on a daily basis. Height and weight were measured without shoes and in light summer school uniform. Timing of the measurements was in mornings or early afternoons. Height measurement was in centimeters (cm) and weight was measured in kilogram (kg) with a range of 0-160 kg. Height and weight were measured to the nearest 0.1 cm and 0.5 kg respectively. Feet were placed together with heels, buttocks and shoulder blades against the stick and head positioned in the Frankfurt horizontal plane.

For each of the sampled classes, demographic information of all officially enrolled students was obtained before data collection, including gender, date of birth, residential address and parental education. Demographic information of students not found on official rosters but currently enrolled in that class was obtained from class teachers. Parental education level was based on the parent with the highest total years of schooling and neighborhood income level was based on the approximate income estimate of child's residential area obtained from the Revenue Department of City District Government Lahore. The study instrument was a structured questionnaire, designed in English. It was pre-tested in the study's intended population and modified accordingly. The questionnaire was tested for reliability (one-week test-retest). It included questions on family and socio-cultural environment including parental working status, number of siblings, number of persons in child's living room and smoking in living space [16,17]. Senior medical students trained in the interviewing techniques interviewed children in the presence of their class teacher (guardian). Each child was asked regarding whether his/her mother works outside or is she a housewife, how many older/younger siblings he/she has, how many persons were living in his/her living room and does anyone smoke in living place? Smoking was defined as smoking tobacco by cigarette, cigar, pipe or hookah. Hookah (water pipe) is a single or multi-stemmed instrument used for smoking tobacco in South Asia.

Quality control measures and good practices included training of data collection team, pre-testing of processes and materials and field monitoring of data collection. Timely availability of the study instruments, meeting of data collection team at the end of everyday to share experiences and submit completed forms, and troubleshooting field problems was ensured. Informed consent statement was printed on the form. Verbal informed consent for the child to participate in the study was taken from class teachers and school heads considering them as guardians. As the study involved no invasive procedure, verbal informed consent was deemed sufficient. The study was approved by the Ethical Review Board of Allama Iqbal Medical College, Lahore. Permissions to conduct the study were granted by the Punjab Departments of Education and Health, and the sampled schools.

### Statistical Analysis

Data were entered and analyzed by manual and computerized checking using SPSS version 18.0 (SPSS Inc. Chicago IL, United States, 2009). Age was calculated to the precise day by subtracting the date of birth from the date of examination. The z-score values for height-for age and BMI-for-age were calculated using the WHO AnthroPlus [18]. Stunting (< -2SD of height-for-age z-score) and thinness (< -2SD of BMI-for-age z-score) were defined using the WHO reference 2007 [19,20]. For international comparison, three grades of thinness were also defined using the International Obesity Task Force (IOTF) cut-offs corresponding to a BMI of 18.5, 17.0 and 16.0 kg/m2 at 18 years of age [21]. Bivariate analysis, using chi-square test as the test of trend, was conducted to compare differences in prevalence of stunting and thinness among the study variables. Crude odds ratios (OR) with 95% confidence interval (CI) were calculated to examine the relationship between stunting and thinness and the study variables by univariate analyses. Multivariate logistic regression was used to quantify the independent predictors of stunting and thinness and adjusted odds ratios (aOR) with 95% CI were obtained. Linear regression was used to explore the predictive power of independent variables in relation to height-for-age and BMI-for-age z-scores as dependent variables. Statistical significance was considered at P < 0.05 and all tests were 2-sided.

## Results

The study included a sample of 1860 primary school children aged 5-12 years. The male-female ratio was 1.11 with 52.5% boys and 47.5% girls. The sample involved 20% children from each grade and 25% children from each area and SES stratum. Seventy-five percent children were urban and 25% were rural. Twenty percent parents were illiterate followed by those educated up to high school (27%), college (28%) and higher (25%). Majority of children had 1-3 siblings (54%) followed by > 3 siblings (44%) and no sibling (1%). Most children (51%) had > 3 persons in living room followed by 1-3 persons (43%) and no person (6%). Smoking in living place was 30%. Most children (49%) lived in middle-income neighborhoods followed by low-income (35%) and high-income (16%) neighborhoods. Median age (range) was 8 (5-12) years and mean age (SD) was 8.49 (1.81) years.

### Stunting (chronic under-nutrition)

Mean (SD) height and mean (SD) height-for-age z-score was 128.4 (11.4) cm and -0.2 (1.3) respectively. Eight percent (95% CI 6.9-9.4) children were stunted while mild stunting (< -1 SD of height-for-age z-score) and severe stunting (< -3 SD of height-for-age z-score) were observed in 18% (95% CI 16.6-20.1) and 1% (95% CI 0.7-1.6) children respectively [Table 1].

**Table 1:** Prevalence of stunting among primary school children in Lahore, Pakistan

Prevalence	n	% (95% CI)	Mean Height (SD) cm	Mean Height-for-age z-score (SD)
Severe stunting (< -3 SD)	21	1.1 (0.7-1.6)	114.8 (12.2)	-3.7 (0.7)
**Stunting (< -2 SD)**	152	8.2 (6.9-9.4)	118.9 (9.6)	-2.6 (0.6)
Mild stunting (< -1 SD)	341	18.3 (16.6-20.1)	123.1 (9.8)	-1.4 (0.3)
Total	1860	100	128.4 (11.4)	-0.2 (1.3)

**Figure 1:**
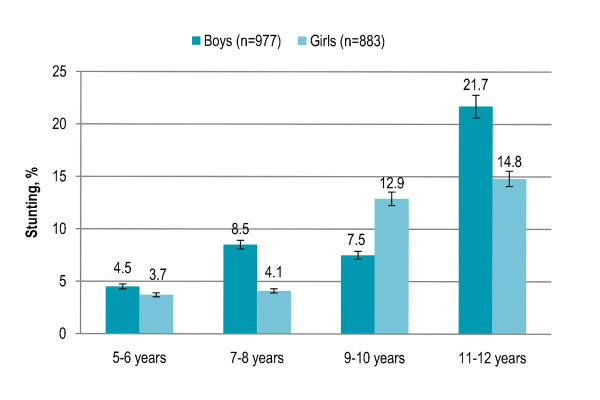
**Age- and gender-specific prevalence (with confidence interval bars) of stunting among primary school children in Lahore, Pakistan**.

**Figure 2:**
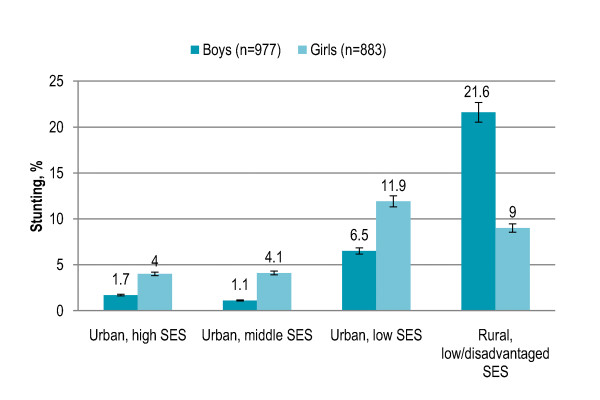
**Gender-specific prevalence (with confidence interval bars) of stunting among primary school children in Lahore, Pakistan by area and socioeconomic status (SES)**.

**Figure 3:**
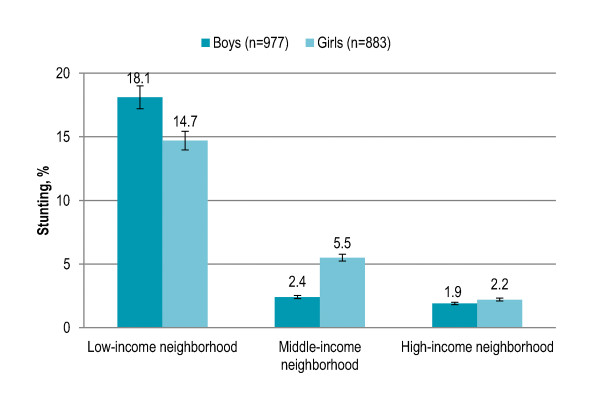
**Gender-specific prevalence (with confidence interval bars) of stunting among primary school children in Lahore, Pakistan by neighborhood income level**.

Gender disparity in overall stunting prevalence was not significant. Stunting among children aged 5-6 years (4%) and 7-8 years (6%) was significantly lower (P < 0.001) as compared to children aged 9-10 years (10%) and 11-12 years (19%). Stunting increased with age among both boys and girls, and the trend was significant (both P < 0.001). More girls were stunted in 9-10 years age group while stunting was higher among boys in all other age groups [Figure 1]. Rural children with low/disadvantaged SES (17%) and urban children with low SES (10%) had significantly higher risk of being stunted (P < 0.001) than urban children with middle SES (2%) or high SES (3%). Among both boys and girls, stunting was significantly higher in rural area and urban area with low SES (boys P < 0.001, girls P = 0.001). In rural area, stunting was higher among boys while girls were more likely to be stunted in urban area [Figure 2]. Children living in low-income neighborhoods (17%) were significantly more likely to be stunted (P < 0.001) than those living in middle-income (4%) and high-income (2%) neighborhoods. Stunting was significantly higher in low-income neighborhoods among both boys and girls (both P < 0.001). Boys were more likely to be stunted in low-income neighborhoods while stunting was higher among girls in middle-income and high-income neighborhoods [Figure 3]. Stunting among children with illiterate parents was 14% that significantly decreased to 4.5% among children with parents having higher education (P < 0.001). Stunting was 4% among children having no siblings that significantly increased to 12% among children having > 3 siblings (P < 0.001). Eleven percent children having > 3 persons in living room were stunted and the proportion significantly decreased to 1% among children with no person in living room (P < 0.001). Smoking in living place was significantly associated with stunting (P < 0.001). [Table 2]

**Table 2:** Socio-demographic correlates of stunting and thinness among primary school children in Lahore, Pakistan

	Total Sample (n = 1860)	Stunted < -2 SD height-for-age (n = 152)		Thin < -2 SD BMI-for-age (n = 188)	
	
Characteristics	n (%)	n (%)	P value	n (%)	P value
**Gender**					
Male	977 (52.5)	84 (8.6)	0.481	99 (10.1)	0.969
Female	883 (47.5)	68 (7.7)		89 (10.1)	
**Age**					
5-6 years (60-83 months)	460 (24.7)	19 (4.1)	< 0.001	52 (11.3)	0.003
7-8 years (84-107 months)	634 (34.1)	40 (6.3)		52 (8.2)	
9-10 years (108-131 months)	579 (31.1)	58 (10.0)		52 (9.0)	
11-12 years (132-155 months)	187 (10.1)	35 (18.7)		32 (17.1)	
**Area and socioeconomic status (SES)**					
Urban, high SES	465 (25.0)	13 (2.8)	< 0.001	23 (4.9)	< 0.001
Urban, middle SES	465 (25.0)	11 (2.4)		33 (7.1)	
Urban, low SES	465 (25.0)	47 (10.1)		65 (14.0)	
Rural, low/disadvantaged SES	465 (25.0)	81 (17.4)		67 (14.4)	
**Neighborhood income**					
Low	651 (35.0)	109 (16.7)	< 0.001	93 (14.3)	< 0.001
Middle	910 (48.9)	37 (4.1)		80 (8.8)	
High	299 (16.1)	06 (2.0)		15 (5.0)	
**Parental education**					
Illiterate	366 (19.7)	51 (13.9)	< 0.001	55 (15.0)	< 0.001
High school	496 (26.7)	60 (12.1)		63 (12.7)	
College	531 (28.5)	20 (3.8)		44 (8.3)	
Higher education	467 (25.1)	21 (4.5)		26 (5.6)	
**Number of siblings**					
No	26 (1.4)	01 (3.8)	< 0.001	02 (7.7)	0.016
1-3	1008 (54.2)	51 (5.1)		84 (8.3)	
> 3	826 (44.4)	100 (12.1)		102 (12.3)	
**Number of persons in child's living room**					
No	116 (6.2)	01 (0.9)	< 0.001	03 (2.6)	0.006
1-3	791 (42.5)	50 (6.3)		74 (9.4)	
> 3	953 (51.2)	101 (10.6)		111 (11.6)	
**Smoking in living place**					
Yes	546 (29.4)	65 (11.9)	< 0.001	56 (10.3)	0.891
No	1314 (70.6)	87 (6.6)		132 (10.0)	

In univariate analyses, significant predictors of stunting included age > 8 years, rural area and urban area with low SES, low-income neighborhoods, lower parental education, crowded housing and smoking in living place. Multivariate logistic regression analysis was adjusted simultaneously for all socio-demographic factors [Table 3]. Age 11-12 years as compared to age 5-6 years (aOR 3.60, 95% CI 1.89-6.88), urban area with low SES as compared to urban area with high SES (aOR 2.58, 95% CI 1.15-5.81) and low-income neighborhoods (aOR 4.62, 95% CI 1.63-13.10) as compared to high-income neighborhoods showed a significant independent association with stunting. In linear regression analysis of all socio-demographic factors significantly associated with stunting (as independent variables) and height-for-age z-score (as dependent variable), high-income neighborhood showed a significant independent positive association while age (in months) showed a significant independent inverse association [Table 4].

**Table 3:** Logistic regression analysis of socio-demographic factors associated with stunting among primary school children in Lahore, Pakistan (n = 1860)

Characteristics	Crude OR (95% CI)	P Value	Adjusted OR (95% CI)	P Value
**Gender**				
Boys	1.13 (0.81-1.57)	0.481	0.91 (0.62-1.34)	0.641
Girls	Reference		Reference	
**Age**				
5-6 years (60-83 months)	Reference		Reference	
7-8 years (84-107 months)	1.56 (0.89-2.74)	0.118	1.68 (0.95-3.00)	0.076
9-10 years (108-131 months)	2.58 (1.52-4.41)	< 0.001	2.94 (1.68-5.16)	< 0.001
11-12 years (132-155 months)	5.35 (2.97-9.62)	< 0.001	3.60 (1.89-6.88)	< 0.001
**Area and socioeconomic status (SES)**				
Urban, high SES	Reference		Reference	
Urban, middle SES	0.84 (0.37-1.90)	0.679	0.72 (0.30-1.74)	0.465
Urban, low SES	3.91 (2.09-7.33)	< 0.001	2.58 (1.15-5.81)	0.025
Rural, low/disadvantaged SES	7.33 (4.02-13.38)	< 0.001	2.49 (0.95-6.53)	0.063
**Neighborhood income**				
Low	9.82 (4.27-22.61)	< 0.001	4.62 (1.63-13.10)	0.004
Middle	2.07 (0.87-4.95)	0.102	1.81 (0.69-4.74)	0.226
High	Reference		Reference	
**Parental education**				
Illiterate	3.44 (2.03-5.83)	< 0.001	0.55 (0.26-1.16)	0.117
High school	2.92 (1.75-4.89)	< 0.001	0.80 (0.41-1.55)	0.507
College	0.83 (0.45-1.55)	0.562	0.61 (0.32-1.19)	0.146
Higher education	Reference		Reference	
**Number of siblings**				
No	Reference		Reference	
1-3	1.33 (0.18-10.03)	0.781	1.07 (0.13-8.73)	0.952
> 3	3.44 (0.46-25.69)	0.228	1.12 (0.13-9.41)	0.915
**Number of persons in child's living room**				
No	Reference		Reference	
1-3	7.76 (1.06-58.72)	0.044	4.49 (0.60-33.65)	0.144
> 3	13.63 (1.88-98.66)	0.010	4.25 (0.56-32.16)	0.161
**Smoking in living place**				
Yes	1.91 (1.36-2.67)	< 0.001	1.33 (0.92-1.93)	0.136
No	Reference		Reference	

**Table 4:** Linear regression analysis of socio-demographic factors associated with height-for-age z-score among primary school children in Lahore, Pakistan (n = 1860)^a^

Characteristics	Regression coefficient (95% CI)	Standard error	P value
Girls	0.09 (-0.03 to 0.20)	0.06	0.135
Age (months)	-0.01 (-0.02 to -0.01)	< 0.01	< 0.001
Area and socioeconomic status (SES)^b^	-0.08 (-0.17 to 0.01)	0.05	0.064
Higher neighborhood income	0.26 (0.14 to 0.38)	0.06	< 0.001
Higher parental education	0.07 (-0.01 to-0.15)	0.04	0.055
Higher number of siblings	-0.01 (-0.14 to 0.12)	0.07	0.900
Higher number of persons in child's living room	-0.10 (-0.20 to 0.01)	0.05	0.064
No smoking in living place	0.02 (-0.10 to 0.15)	0.07	0.712

^a^R^2 ^for model = 0.111

^b^Area and SES strata: 1. Urban with high SES, 2. Urban with middle SES, 3. Urban with low SES, and 4. Rural with low/disadvantaged SES

### Thinness (acute under-nutrition)

Mean (SD) BMI and mean (SD) BMI-for-age z-score was 20.7 (5.0) kg/m^2 ^and -0.3 (1.5) respectively. Ten percent (95% CI 8.7-11.5) children were thin while mild thinness (< -1 SD of BMI-for-age z-score) and severe thinness (< -3 SD of BMI-for-age z-score) were observed in 23% (95% CI 20.8-24.6) and 3% (95% CI 2.1-3.6) children respectively [Table 5]. According to the IOTF cut-offs, thinness grade 1 was observed in 1.2% (95% CI 0.8-1.8, n = 22) children and thinness grades 2 and 3 were observed in only four and five children respectively.

**Table 5:** Prevalence of thinness among primary school children in Lahore, Pakistan

Prevalence	n	% (95% CI)	**Mean BMI (SD) kg/m**^**2**^	Mean BMI-for-age z-score (SD)
Severe thinness (< -3SD)	52	2.8 (2.1-3.6)	14.9 (1.9)	-4.0 (1.3)
**Thinness (< -2 SD)**	188	10.1 (8.7-11.5)	16.1 (2.0)	-2.8 (1.0)
Mild thinness (< -1 SD)	422	22.7 (20.8-24.6)	17.8 (2.1)	-1.4 (0.3)
Total	1860	100	20.7 (5.0)	-0.3 (1.5)

**Figure 4:**
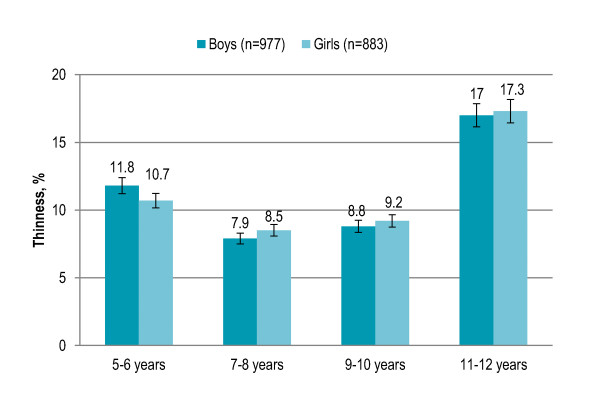
**Age- and gender-specific prevalence (with confidence interval bars) of thinness among primary school children in Lahore, Pakistan**.

**Figure 5:**
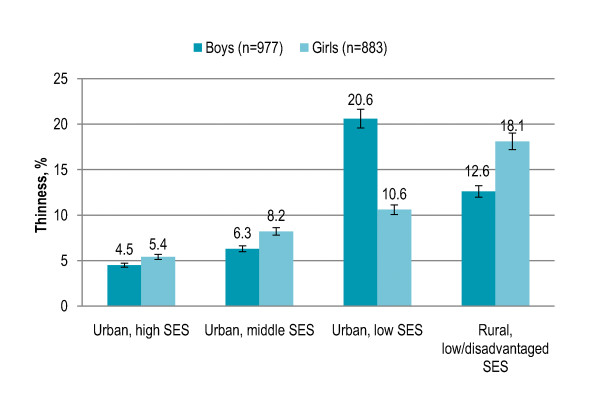
**Gender-specific prevalence (with confidence interval bars) of thinness among primary school children in Lahore, Pakistan by area and socioeconomic status (SES)**.

**Figure 6:**
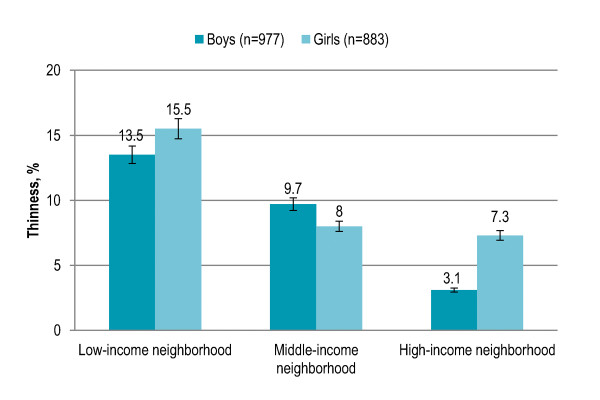
**Gender-specific prevalence (with confidence interval bars) of thinness among primary school children in Lahore, Pakistan by neighborhood income level**.

Gender showed no significant association with overall thinness prevalence. Children aged 5-6 years (11%) and 7-8 years (8%) were significantly less likely to be thin (P = 0.003) as compared to children aged 9-10 years (9%) and 11-12 years (17%). Thinness was 12% among boys aged 5-6 years that slightly decreased among boys aged 7-10 years (8-9%) and peaked among boys aged 11-12 years (17%), and the trend was significant (P = 0.034). Thinness was not significantly associated with age among girls [Figure 4]. Children living in rural area with low/disadvantaged SES (14.4%) and urban area with low SES (14%) had significantly higher risk of being thin (P < 0.001) than children living in urban area with middle SES (7%) or high SES (5%). Among both boys and girls, thinness was significantly higher in rural area and urban area with low SES (boys P < 0.001, girls P = 0.001). In rural area and urban area with middle SES and high SES, thinness was higher among girls while boys were more likely to be thin in urban area with low SES [Figure 5]. Living in low-income neighborhoods (14%) significantly increased the risk of thinness (P < 0.001) as compared to living in middle-income (9%) or high-income (5%) neighborhoods. Among both boys and girls, thinness was significantly higher in low-income neighborhoods (boys P = 0.001, girls P = 0.003). Boys were more likely to be thin in middle-income neighborhoods while thinness was higher among girls in low-income and high-income neighborhoods [Figure 6]. Thinness among children with illiterate parents was 15% that significantly decreased to 6% among children with parents having higher education (P < 0.001). Children having more siblings (P = 0.016) and more persons in living room (P = 0.006) were significantly more likely to be thin. Smoking in living place was not significantly associated with thinness. [Table 2]

In univariate analyses, significant predictors of thinness included rural area and urban area with low SES, low-income and middle-income neighborhoods, lower parental education and crowded housing. In multivariate logistic regression analysis adjusted simultaneously for all socio-demographic factors, only urban area with low SES as compared to urban area with high SES (aOR 2.28, 95% CI 1.21-4.30) showed a significant independent inverse association with thinness [Table 6]. In linear regression analysis of all socio-demographic factors significantly associated with thinness (as independent variables) and BMI-for-age z-score (as dependent variable), only rural area with low/disadvantaged SES showed a significant independent inverse association [Table 7].

**Table 6:** Logistic regression analysis of socio-demographic factors associated with thinness among primary school children in Lahore, Pakistan (n = 1860)

Characteristics	Crude OR (95% CI)	P Value	Adjusted OR (95% CI)	P Value
**Gender**				
Boys	1.01 (0.74-1.36)	0.969	1.04 (0.75-1.45)	0.805
Girls	Reference		Reference	
**Age**				
5-6 years (60-83 months)	Reference		Reference	
7-8 years (84-107 months)	0.70 (0.47-1.05)	0.085	0.78 (0.52-1.18)	0.247
9-10 years (108-131 months)	0.77 (0.52-1.16)	0.216	0.93 (0.61-1.41)	0.727
11-12 years (132-155 months)	1.62 (1.01-2.61)	0.048	1.61 (0.95-2.72)	0.078
**Area and socioeconomic status (SES)**				
Urban, high SES	Reference		Reference	
Urban, middle SES	1.47 (0.85-2.54)	0.170	1.34 (0.73-2.47)	0.350
Urban, low SES	3.12 (1.91-5.12)	< 0.001	2.28 (1.21-4.30)	0.011
Rural, low/disadvantaged SES	3.24 (1.98-5.30)	< 0.001	1.73 (0.79-3.77)	0.171
**Neighborhood income**				
Low	3.16 (1.80-5.55)	< 0.001	1.29 (0.62-2.65)	0.498
Middle	1.83 (1.03-3.22)	0.038	1.21 (0.63-2.30)	0.568
High	Reference		Reference	
**Parental education**				
Illiterate	3.00 (1.84-4.89)	< 0.001	1.65 (0.86-3.19)	0.135
High school	2.47 (1.53-3.97)	< 0.001	1.52 (0.87-2.67)	0.141
College	1.53 (0.93-2.53)	0.095	1.35 (0.81-2.26)	0.250
Higher education	Reference		Reference	
**Number of siblings**				
No	Reference		Reference	
1-3	1.09 (0.25-4.70)	0.907	0.86 (0.19-3.89)	0.846
> 3	1.69 (0.39-7.26)	0.480	0.90 (0.20-4.17)	0.896
**Number of persons in child's living room**				
No	Reference		Reference	
1-3	3.89 (1.21-12.54)	0.023	2.87 (0.87-9.39)	0.082
> 3	4.97 (1.55-15.86)	0.007	2.69 (0.81-8.92)	0.105

**Table 7:** Linear regression analysis of socio-demographic factors associated with BMI-for-age z-score among primary school children in Lahore, Pakistan (n = 1860)^a^

Characteristics	Regression coefficient (95% CI)	Standard error	P value
Girls	-0.08 (-0.21 to 0.05)	0.07	0.248
Age (months)	0.01 (-0.01 to 0.01)	< 0.01	0.285
Area and socioeconomic status (SES)^b^	-0.39 (-0.49 to -0.29)	0.05	< 0.001
Higher neighborhood income	0.04 (-0.10 to 0.17)	0.07	0.587
Higher parental education	0.06 (-0.02 to 0.15)	0.04	0.142
Higher number of siblings	-0.08 (-0.23 to 0.06)	0.07	0.252
Higher number of persons in child's living room	-0.07 (-0.18 to 0.05)	0.06	0.269

^a^R^2 ^for model = 0.141

^b^Area and SES strata: 1. Urban with high SES, 2. Urban with middle SES, 3. Urban with low SES, and 4. Rural with low/disadvantaged SES

## Discussion

Prevalence estimates and socio-demographic correlates of stunting and thinness among Pakistani primary school children aged 5-12 years are presented. This was the first study in Pakistan to report prevalence estimates for under-nutrition among school-aged children based on the WHO reference 2007. We could find only three studies in previous literature with a representative sample that report prevalence of stunting (14-17%) and wasting (25-32%) among school-aged children in Pakistan using the World Health Organization/National Centre of Health Statistics (WHO/NCHS) reference [11-13]. Prevalence of stunting and underweight among urban school-aged children in Pakistan has been reported as 17% and 30% respectively in 1990-94 (National Health Survey of Pakistan, n = 1670) that decreased to 14% and 27% respectively in 2004-05 (Karachi survey, n = 1381) [13]. Significant improvement in the nutritional status was observed in the present study with stunting and thinness prevalence of 8% and 10% respectively; however, the inequities were more marked with the highest prevalence being among the poor and socially disadvantaged population. Thinness prevalence by the IOTF cut-offs was very low and grade 1 thinness corresponded to severe thinness by the WHO reference.

Both stunting and thinness were not significantly associated with gender. However, gender differences were more marked in low socioeconomic groups with boys being more undernourished, consistent with previous literature [22]. Stunting and thinness showed a significant increasing trend with age and older age was the independent predictor of stunting. Progression of height deficit with increasing age had been reported previously in Pakistan and elsewhere [8,23-27].

Rural children, urban children with low SES and those living in low-income neighborhoods were at higher risk of being stunted and thin. Poverty and low socioeconomic status had a more detrimental effect on linear growth than on body weight, in line with previous literature [4,28-30]. Higher stunting and wasting among rural children had been reported in Pakistan and elsewhere [12,31-36]. Economic inequality is an independent determinant for childhood under-nutrition and a number of studies have illustrated that the poor children tend to be at higher risk of being undernourished and having restricted growth [11,25,26,28,31,37-45]. Countries with a greater degree of economic inequality tend to have a poor health status than countries with more economic equality [46]. The developing countries remain vulnerable to food insecurity, poor access to health services, under-nutrition and increased morbidity and mortality, and the health and nutritional benefits from economic growth tend to be concentrated only among the economically advantaged population groups [9,28,31,38,47].

Stunting and thinness among children with illiterate parents was significantly higher as compared to children with parents having higher education. Parental education had been identified in other populations as a predictor of under-nutrition [25,26,31,41-44,48-50]. Poorest population segments are the least educated that increases the gap between the richest and the poorest. Stunting and thinness were significantly higher in children having more siblings and living in crowded houses, both these factors are indirect predictors of lower socioeconomic status. Smoking in living place was significantly associated with being stunted while the association was not significant for thinness. Although the effect did not remain significant in adjusted analysis but it corresponded with previous studies that report higher risk of childhood under-nutrition with smoking [51-53].

Integrated nutrition programs in the developing countries have had a substantial impact through a combination of targeted interventions involving fields of health, water supply, sanitation, food and education [54-57]. A school-based food program focused on the poorest population groups can be considered in the developing countries where a child can be fed in school just for about US$ 34 annually and school-based feeding programs can provide a launching pad for preventive programs including immunization, growth monitoring, deworming and targeted micronutrient supplementation [57]. In Pakistan, School Health and Nutrition Supervisors working in the National Maternal, Newborn, and Child Health (MNCH) Program can be used for this purpose.

Cross-sectional nature of the study should be considered when interpreting the findings. Although data collection followed a standard protocol, digital scales were not used. Variability in the data ascertainment may have introduced error into the prevalence estimates; however, we do not anticipate large or systematic differences. In Pakistan, estimates for neighborhood income level were not available in the census and statistical data; therefore, the division in low-, middle- and high-income neighborhoods was based on the approximate estimates by the Revenue Department of City District Government Lahore. The findings can be generalized to urban South Asian primary school children, who share the same genetic and environmental factors with the sample.

## Conclusions

Relatively low prevalence of stunting and thinness depicted an improvement in the nutritional status of school-aged children in Pakistan. However, the inequities between the poorest and the richest population groups were marked with significantly higher prevalence of stunting and thinness among the rural and the urban poor, the least educated, the residents of low-income neighborhoods and those having crowded houses. An increasing trend with age was observed in prevalence of stunting and thinness. Smoking in living place was associated with stunting. Findings suggest the need to implement evidence-based child health policy and strategies, prioritizing the poor and socially disadvantaged population. Future national nutritional surveys in the developing countries ought to consider including school-aged children and nutritional component of the child health programs need to be strengthened.

## Competing interests

The authors declare that they have no competing interests.

## Authors' contributions

All authors contributed significantly in all phases of the study in accordance with uniform requirements established by the International Committee of Medical Journal Editors. All authors read and approved the final manuscript.

## Pre-publication history

The pre-publication history for this paper can be accessed here:

http://www.biomedcentral.com/1471-2458/11/790/prepub
